# Active Stat3 is required for survival of human squamous cell carcinoma cells in serum-free conditions

**DOI:** 10.1186/1476-4598-5-15

**Published:** 2006-04-07

**Authors:** Weihong Yin, Satish Cheepala, Jennifer N Roberts, Keith Syson-Chan, John DiGiovanni, John L Clifford

**Affiliations:** 1 Department of Biochemistry and Molecular Biology, Louisiana State University Health Science Center School of Medicine in Shreveport and Feist-Weiller Cancer Center, Shreveport, LA 71130, USA; 2 Department of Carcinogenesis, University of Texas-M.D. Anderson Cancer Center, Science Park Research Division, PO Box 389, Smithville, TX 78957, USA

## Abstract

**Background:**

Squamous cell carcinoma (SCC) of the skin is the most aggressive form of non-melanoma skin cancer (NMSC), and is the single most commonly diagnosed cancer in the U.S., with over one million new cases reported each year. Recent studies have revealed an oncogenic role of activated signal transducer and activator of transcription 3 (Stat3) in many human tumors, especially in those of epithelial origin, including skin SCC. Stat3 is a mediator of numerous growth factor and cytokine signaling pathways, all of which activate it through phosphorylation of tyrosine 705.

**Results:**

To further address the role of Stat3 in skin SCC tumorigenesis, we have analyzed a panel of human skin-derived cell lines ranging from normal human epidermal keratinocytes (NHEK), to non-tumorigenic transformed skin cells (HaCaT), to highly tumorigenic cells (SRB1-m7 and SRB12-p9) and observed a positive correlation between Stat3 phosphorylation and SCC malignancy. We next determined the role of Stat3 activity in cell proliferation and viability under serum-free culture conditions. This was accomplished by suppressing Stat3 activity in the SRB12-p9 cells through stable expression of a dominant negative acting form of Stat3β, which contains a tyrosine 705 to phenylalanine mutation (S3DN). The S3DN cells behaved similar to parental SRB12-p9 cells when cultured in optimal growth conditions, in the presence of 10% fetal calf serum. However, unlike the SRB12-p9 cells, S3DN cells underwent apoptotic cell death when cultured in serum-free medium (SFM). This was evidenced by multiple criteria, including accumulation of sub-G1 particles, induced PARP cleavage, and acquisition of the characteristic morphological changes associated with apoptosis.

**Conclusion:**

This study provides direct evidence for a role for Stat3 in maintaining cell survival in the conditions of exogenous growth factor deprivation produced by culture in SFM. We also propose that delivery of the S3DN gene or protein to tumor cells could induce apoptosis and/or sensitize those cells to the apoptotic effects of cancer therapeutic agents, raising the possibility of using S3DN as an adjunct for treatment of skin SCC.

## Introduction

Non-melanoma skin cancer (NMSC) is the most common cancer in the U.S., with over a million new cases of the two most common forms, squamous and basal cell carcinoma, anticipated in 2004 [1]. The more clinically aggressive form, squamous cell carcinoma (SCC) [2], has been increasing in incidence since the 1960s at annual rates from 4% to as much as 10% in recent years [3]. About 95% of skin SCC cases are diagnosed at an early stage and are easily controlled. Unlike early stage SCC, advanced SCC is aggressive, often resistant to local therapy, requires repeated surgical resections and courses of radiotherapy, and accounts for approximately 2000 U.S. deaths each year [1,4]. Advanced disease- and treatment-related morbidity have a profound impact on patients' quality of life, frequently producing cosmetic deformity, loss of function, and psychosocial problems. Improved control of advanced skin SCC is clearly necessary and will rely on a thorough understanding of the molecular basis for skin SCC progression.

Signal transducers and activators of transcription (Stat) proteins, a family of latent cytoplasmic transcription factors, are expressed in many cell types and, in response to a wide variety of extracellular polypeptides, regulate the transcription of a broad spectrum of genes that are critically involved in cytokine signaling [5], cell proliferation and development [6], and tumorigenesis [7-9]. Upon binding of extracellular ligands, cell surface receptors oligomerize and activate associated Janus kinases (JAKs), which in turn phosphorylate Stats on a single critical tyrosine residue located adjacent to an -SH2 (src homology domain 2) domain. The Stats then dimerize via reciprocal -SH2 domain phosphorylation site interactions and translocate to the nucleus where they regulate gene expression by direct DNA binding or by associating with other transcription factors [10,11]. The activity of Stats can be abolished by mutation of this critical tyrosine [12,13].

Among the seven known members of mammalian Stat family, Stat3 has been most strongly implicated in tumorigenesis [7-9]. Elevated levels of Stat3 activity have been observed in a number of human cancers and cancer cell lines [9]. In cancers of epithelial origin, Stat3 is constitutively activated in head and neck squamous cell carcinoma (HNSCC) [14,15], breast cancer cell lines [16,17], ovarian cancer cell lines [18], and lung cancer cell lines [19]. In particular, Stat3 plays a critical role in the development of skin cancer [20]. In an experimental two-stage mouse skin chemical carcinogenesis model it has been shown that Stat3 is constitutively activated in skin tumors [21], and that activated Stat3 is indispensable for both the initiation and the promotion stages of epithelial carcinogenesis [22]. The critical role of Stat3 in skin tumor development was further supported by data obtained from a transgenic mouse model in which a constitutively active mutant of Stat3 called Stat3C (7), was expressed in skin under the control of the keratin-5 promoter [23]. These mice have a skin phenotype closely resembling psoriasis in humans and, when subjected to the two-stage skin chemical carcinogenesis protocol, rapidly developed carcinomas, bypassing the papilloma stage that normally takes place in this model [[23], Chan et al, submitted].

Apoptosis or programmed cell death, is mediated through two major pathways, the extrinsic and intrinsic [24,25]. The extrinsic pathway is primarily triggered by the binding of extra-cellular death ligands (e.g. TNFα, TRAIL and FasL) to their cognate membrane death receptors. The intrinsic pathway is often initiated by cellular stresses such as withdrawal of survival factors, direct DNA damage (e.g. UV exposure, cytotoxic drugs), and is characterized by the disruption of mitochondrial membrane integrity, an event regulated by Bcl-2 protein family members [26,27]. There are more than 20 known members of the Bcl-2 family which, based on their functions in regulating apoptosis, can be divided into an anti-apoptotic 'Bcl-2-like' group, (Bcl-2, Bcl-XL, Bcl-w, Bfl-1/A1 and Mcl-1 etc) and a pro-apoptotic group (Bax, Bak, Bok, Bcl-Xs, Bad, Bid, Bik/Nbk, Bim, Hrk, Bmf, Noxa and Puma etc). It has been reported that Stat3 can regulate transcription of several Bcl-2 family proteins, such as Bcl-2 and Bcl-xL [28-31], Bax [32], and Mcl-1 [33-35].

At early stages of tumor development, tumor cells often have to face and survive harsh physiological micro-environments, such as lack of nutrition and/or blood supply, survival factor insufficiency, and hypoxia, which generally lead to apoptosis in normal cells [36]. In fact, it has been well accepted that one of the six hallmarks of tumor cells is the reduced or complete loss of dependence on exogenous growth factor stimulation for survival and proliferation [37]. Stats are the first family of transcription factors found to be directly activated upon growth factor receptor stimulation. Stat3 is thought to confer protection against apoptosis in many transformed or tumor cells. Several studies in which Stat3 activity is either blocked by anti-sense oligonucleotides, small interfering RNA or expression of dominant negative Stat3 isoforms [14,32,38-40], or elevated by expression of Stat3C [41], have shown an inverse correlation between Stat3 activity and induced apoptosis.

In this study we have examined the activity of Stat3 in several human skin-derived cell lines, ranging from non-transformed to highly malignant, and observed a positive correlation between malignancy and constitutive Stat3 phosphorylation. In addition, we have generated human skin SCC cell lines with reduced Stat3 activity by stably expressing a dominant negative acting form of Stat3β, hereafter referred to as S3DN [42-44]. The S3DN cells, unlike the parental SRB12-p9 cells, undergo apoptosis in the conditions of exogenous growth factor deprivation produced by culture in serum free medium (SFM).

## Results

### Constitutive phosphorylation of Stat3 positively correlates with malignancy of human skin SCC cell lines

**Figure 1:**
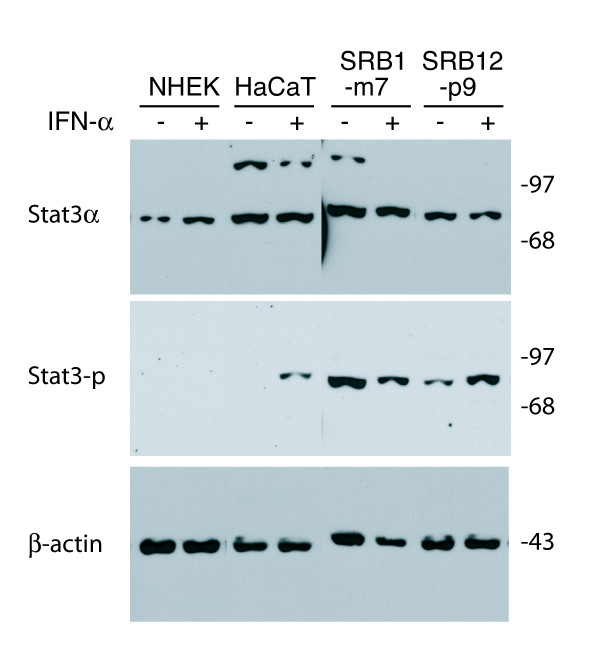
Stat3 phosphorylation correlates with human skin SCC malignancy. NHEK cells were grown for 48 hours in KGM-2 growth media and HaCaT, SRB12-p9 and SRB1-m7 cells were grown for 48 hours in normal growth media containing 0.5% serum, followed by culture for 2 hours in SFM and then treated for 30 min with 100 IU/ml IFN-α. Whole cell extracts were purified, subjected to Western blotting and sequentially probed with antibodies specific to Stat3α, tyrosine 705 phosphorylated Stat3α (Stat3-p) (middle panel), and β-actin as a control for well loading.

Stat3 transcriptional activity can be induced by the phosphorylation of a single tyrosine residue (Tyr705) by receptor associated Janus kinases. Increased Stat3 activation is often associated with either elevated constitutive levels of Stat3 protein or increased Stat3 tyrosine phosphorylation [14]. To further elucidate the role of Stat3 in skin cancer development, we first examined the Stat3 status in NHEKs, non-tumorigenic, spontaneously transformed keratinocyte cells (HaCaT) and two aggressive skin SCC cell lines (SRB1-m7 and SRB12-p9). In order to avoid detecting a high level of background Stat3 phosphorylation, the HaCaT and SRB cells were switched from their normal growth in medium which contains 5 and 10% FCS, respectively, to medium containing 0.5% serum for two days, followed by an additional two hours culture in SFM. We have previously reported that low concentrations of IFN-α efficiently induce Stat3 phosphorylation in SRB12-p9 cells [45]. In order to compare the inducibility of Stat3 phosphorylation between normal, premalignant and malignant skin cells, they were treated with 100 international units (IU)/ml IFN-α for 30 min, and then whole cell protein was extracted and subjected to western blot probing with a Stat3α- or a Stat3 phospho-tyrosine 705-specific antibody. All cell lines expressed comparable steady state levels of Stat3α protein with or without IFN-α treatment (Fig. 1, upper panel). However, in the absence of IFN-α treatment, phosphorylated Stat3α (phospho-Stat3α) was only observed in the tumorigenic skin SCC cell lines (Fig. 1, lanes labeled SRB1-m7 and SRB12-p9, middle panel). Treatment with IFN-α, which has been previously shown to induce Stat3 phosphorylation and DNA binding [44], induced phosphorylation in the HaCaT cells, but not in the NHEK cells (Fig. 1 middle panels, NHEK and HaCaT lanes). **The upper band in lanes 3–5 is non-specific and is sometimes observed with this antibody**.

### Establishment of cell lines stably expressing a dominant negative form of Stat3 (S3DN)

**Figure 2:**
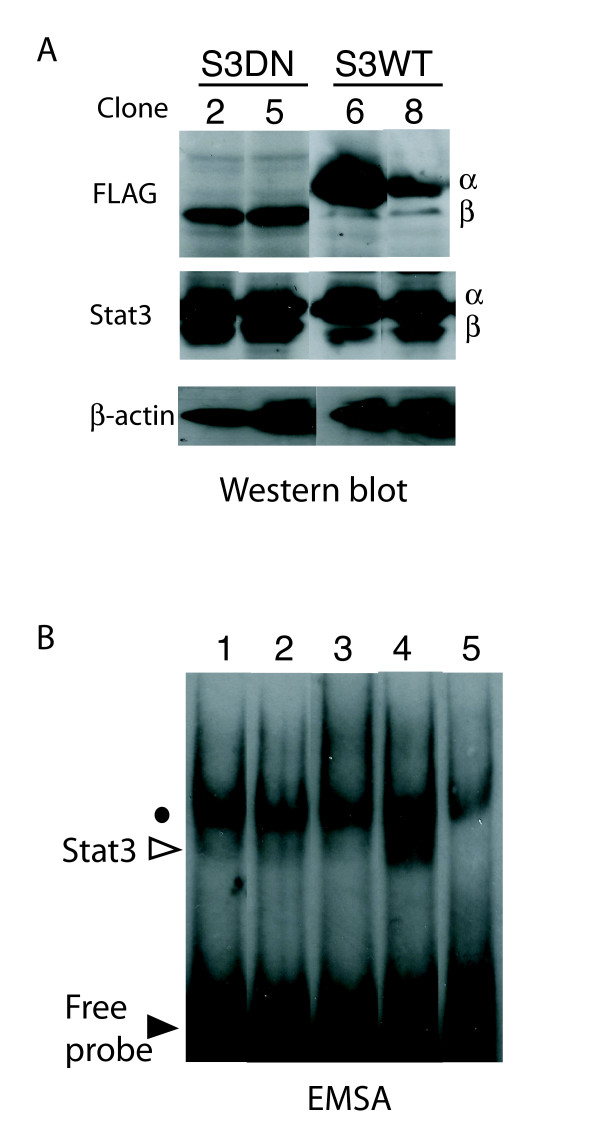
Establishment of cell clones stably expressing S3DN and S3WT proteins. (A) SRB12-p9 cells were transfected with expression vectors as described in materials and methods. Whole cell extracts from cell clones (number shown above each lane) were subjected to Western blotting and probed sequentially with antibodies to the FLAG octapeptide, Stat3 (recognizing both α and β isoforms), and β-actin. (B) SRB12-p9, Neo, S3DN2 and S3WT6 cells (lane 1, 2, 3 and 4 respectively) were serum-starved for 2 days. Nuclear extracts were prepared and the EMSA assay was performed as described in Materials and Methods. In lane 5 nuclear extract from S3WT6 was pre-incubated with excess cold (unlabeled) Stat3 probe prior to the addition of labeled probe. Open arrow, expected location for Stat3α homodimer; closed arrow, unbound probe; dot, non-specific band. Results are representative of three separate experiments.

To explore the role of Stat3 in skin cell malignancy, we over-expressed both the wild type Stat3α and a dominant negative form of Stat3β in one of the tumorigenic SCC cell lines, SRB12-p9. It has been reported that Stat3β, a naturally occurring Stat3 splice variant that has a truncated C-terminus, can function as a dominant negative form of Stat3 and inhibit its transcriptional activity [42,43]. It was subsequently shown that substituting the critical Jak kinase tyrosine phosphorylation site with phenylananine generated a form of Stat3 that could block DNA binding by all endogenous forms of Stat3 (Stat3β-Y705F) [44]. We established SRB12-p9 cell clones expressing either the FLAG-tagged wild type Stat3α protein (S3WT) or FLAG-tagged Stat3β-Y705F (S3DN) (Fig. 2A). Over-expressing the S3WT resulted in higher Stat3 DNA binding activity than in parental cells as determined by EMSA (Fig. 2B, compare lane 4 with lane 1, white triangle), while the DNA binding activity in cells expressing the S3DN was reduced (Fig. 2B, compare lane 3 to lanes 1 and 2). The specificity of DNA binding was confirmed by the elimination of the shifted band upon addition of excess unlabelled Stat3 probe (Fig. 2B, compare lane 5 with lane 4). We note that the EMSA band intensities in Fig. 2B are lower than those typically detected for lysates of cells transiently transfected with expression constructs for Stats (see [44]). In the present study we assayed for endogenous and stably expressed Stat3 isoforms, which are less abundant than in transiently transfected cells.

### Stat3 activity is required for cell survival in SFM

**Figure 3:**
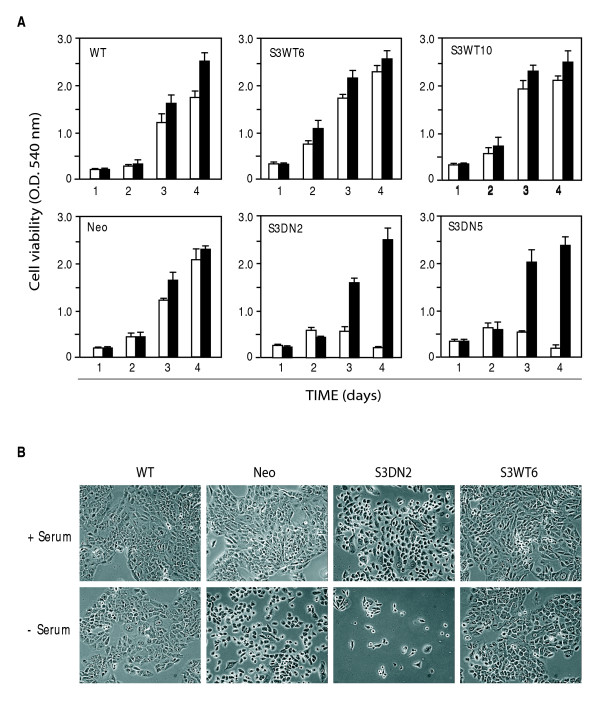
Stat3 activity is required for survival in SFM. (A) SRB12-p9, Neo and two representative independent S3WT (S3WT6, S3WT10) and S3DN clones (S3DN2, S3DN5) were cultured for the indicated times in 10% FCS-containing media or SFM and cell viability was determined by the MTT assay (see materials and methods). White and black bars indicate the mean absorbance at 540 nm for cells grown in SFM or 10% FCS-containing media, respectively. Error bars indicate SEM for triplicate cultures. Results shown are representative of >4 assays that included 2 independent S3WT and S3DN cell clones. (B) SRB12-p9, Neo, S3DN2 and S3WT6 cells were cultured for 5 days in 10% FCS-containing media or SFM (upper and lower rows, respectively). Results shown are representative of 3 independent S3DN and S3WT cell clones from 4 separate experiments. Cells were photographed on a phase contrast microscope (100×).

Stressful physiological conditions, such as a shortage of nutrients or growth factors, often result in apoptotic cell death in non-transformed cells but not in transformed cells. The S3WT and S3DN cell lines allowed determination of the role of Stat3 on cell viability during culture in SFM, a well-established experimental stress condition. Cells were grown in the presence or absence of FCS for 4 days and cell viability measured by the MTT assay at 1 day intervals. Doubling times for cells growing at a subconfluent density (between days 1 and 3) were calculated based on the viability data shown in Fig. 3A. In the standard 10% FCS-containing medium there was no major difference in the cell viability and calculated doubling times for parental SRB12-p9 cells and control SRB12-p9 cells stably transfected with the empty pSG5 expression vector and the pKJ1 neomycin resistance vector (Neo) (Fig. 3A, black bars and Table 1). The S3WT cells (represented by clones 6 and 10) had an increase in doubling time of approximately 2 hours and one of the S3DN cell lines (S3DN5) was increased by 3 hours, indicating no correlation between Stat3 expression pattern and doubling time in standard media (Fig. 3A and Table 1). However, in SFM (Fig. 3A, white bars) the two S3DN cell lines showed no increase in cell number between days 2 and 3 and by the 4^th ^day had a reduction in cell viability, indicating cell death. Visual inspection revealed that S3DN cells cultured in SFM became rounded and detached from the plate, two events that typically accompany apoptotic cell death (Fig. 3B, compare upper and lower S3DN2 panels). In contrast, the SRB12-P9, Neo and S3WT cells showed only a slight reduction in cell viability compared to cells grown in FCS-containing medium (Fig. 3A compare white bars to black bars and table 1). From this data we conclude that Stat3 activity is required for SCC cell survival in SFM. This response was consistently observed for SRB12-p9 cells and multiple independent Neo (n = 2), S3DN (n = 3) and S3WT (n = 3) cell clones, for 4 experiments.

**Table 1:** Expression of wild type Stat3α (S3WT column) and dominant negative Stat3β (S3DN column) protein does not correlate with cell doubling time. Cell doubling time was calculated as described in Materials and Methods using the proliferation data shown in Fig. 3A. + symbol refers to the approximate level of S3WT or S3DN protein, as determined by Western blotting. N/A: not applicable.

			Doubling Time	
Cell line	Relative protein level		Day 1 through Day 3 ime	
	S3WT	S3DN	10% FCS	-FCS
SRB12-p9	+	-	15.6	18.6
P9 neo	+	-	15.4	17.9
S3DN2	+	++	15.3	N/A
S3DN5	+	++	18.4	N/A
S3WT6	+++	-	17.4	20
S3WT10	+	-	17.4	18.6

### Expression of S3DN protein causes apoptosis in cells grown in SFM

**Figure 4:**
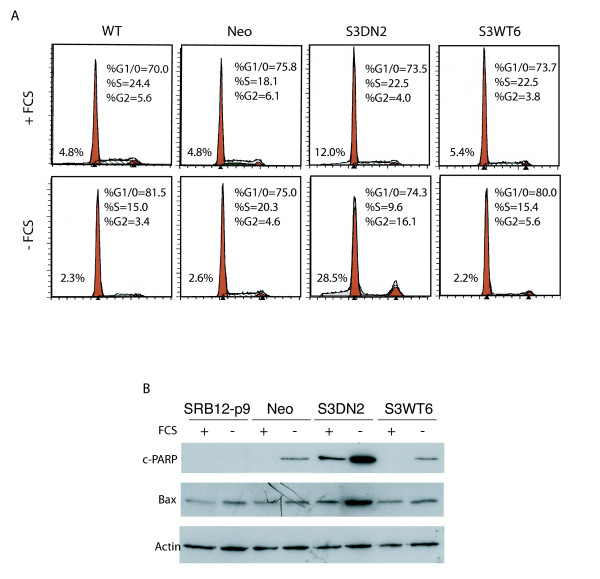
Expression of S3DN protein leads to apoptosis in SFM. SRB12-p9, Neo, S3DN2 and S3WT6 cells were cultured in 10% FCS-containing media or SFM for 4 days. (A) Cells and floating material were collected, fixed in 70% ethanol, stained with propidium iodide and analyzed by flow cytometry. The percentage of cells in the G1/0, S and G2 phases of the cell cycle are indicated for each cell line and treatment condition. Numbers to the left of the G1/0 peak indicate the percentage of sub-G1/0 particles from the total population of gate particles (excluding clumped cells). (B) Whole cell extracts were resolved on an SDS polyacrylamide gel, transferred to nitrocellulose and sequentially probed with antibodies specific for c-PARP, Bax and β-actin. Results shown in A and B are representative of 2 Neo and 3 S3DN and S3WT clones, in 2 independent experiments.

The reduced viability of the S3DN cells grown in SFM could be due to either cell death alone or to a combination of cell death and reduced proliferation. There is abundant evidence for a role for Stat3 both in regulating proliferation and in suppressing apoptotic cell death [7-9]. In order to determine whether expression of the S3DN or S3WT proteins could also influence proliferation, we compared the cell cycle profiles of the SRB12-p9, Neo, S3DN and S3WT cells. Cells were cultured under conditions identical to that shown in Fig. 3B and their cell cycle profiles determined by flow cytometry. In FCS-containing media all cell populations had similar percentages in the G1, S and G2 phases of the cell cycle (Fig. 4A, upper panels). When grown in SFM for 4 days several small differences were observed. SRB12-P9, S3DN2 and S3WT6 cells showed reductions in the percentage of cells in S phase, **the S3DN2 cells showed an increase in the percentage of cells in the G2 phase**, and SRB12-P9 and S3WT6 cells showed an increase in the percentage of cells in the G1 phase (Fig. 4A, -FCS panels). However the most prominent difference was the greater number of DNA containing particles in the sub-G1 size range observed for the S3DN2 cells in both FCS-containing media (12% of total particle count compared to approximately 5% for the other cell lines) and in SFM (28.5% compared to 2–3% for the other cells, Fig 4A). The presence of sub-G1 particles is a hallmark of apoptosis for cultured cells. This finding was reproducible for multiple Neo, S3DN and S3WT cell clones and is consistent with the results shown in Fig. 3A and 3B, strongly suggesting that S3DN expression primarily affects the control of apoptosis and not proliferation in this model.

In order to further confirm that the loss of cell viability of the S3DN cells grown in SFM is due to increased apoptosis, we assayed for the appearance of c-PARP protein, the cleaved from of PARP, in cells treated as in Fig. 4A. PARP cleavage facilitates irreversible cellular disassembly and provides a highly sensitive indicator of apoptosis [46]. The appearance of c-PARP was most pronounced in the S3DN2 cells grown in SFM, as determined by western blotting with a c-PARP-specific antibody (Fig. 4B, upper panel). A low level of c-PARP was detectable in S3DN2 cells grown in FCS-containing media, as well as in Neo and S3WT6 cells grown in SFM (Fig. 4B, upper panel).

It has been reported in other cell culture systems and animal models that Stat3 regulates apoptosis via modulation of transcription of several of the Bcl-2 family proteins, including Bcl-2, Bcl-xL, Bax and Mcl-1 [28-35]. Here we observe enhanced Bax protein expression for S3DN2 grown in SFM, but not for the other cells, suggesting a possible role for Bax in this apoptotic effect (Fig. 4B, middle panel). As in 4A, these results are representative of several Neo, S3DN and S3WT cell clones in at least 2 independent experiments per clone.

## Discussion

Elevated Stat3 activity has been observed in numerous spontaneous and experimentally established mammalian cancers, demonstrating a critical role in tumorigenesis [7-9,17]. In this study we provide direct evidence that Stat3 activity, as indicated by phosphorylation at tyrosine 705, positively correlates with malignancy in human skin-derived cell lines. Suppression of Stat3 activity, through forced expression of the S3DN protein, in human skin SCC cells blocks their growth factor- and/or other serum factor-independence, a major characteristic of malignancy.

Recent studies have provided convincing evidence for a critical role for Stat3 in every stage of mouse skin cancer development, from promoting the survival of initiated cells to conferring late-stage malignant characteristics such as enhanced motility and invasiveness [21,22]. In parallel with these studies we sought to develop a human skin SCC model in which Stat3 activity is stably suppressed, in order to assess the contribution of activated Stat3 to the malignant phenotype in human disease. The SRB12-p9 cell line was originally derived from an aggressive skin SCC tumor. These cells were chosen for stable transfection of the S3DN protein because, in addition to having constitutive and IFN-α inducible Stat3 phosphorylation, they are highly tumorigenic upon subcutaneous injection into nude mice and can be readily transfected and selected for stable gene expression [45].

The S3DN protein is a unique Stat3 blocking reagent. It consists of Stat3β, τhe short alternative splice product of the Stat3 gene, bearing a point mutation at the critical tyrosine 705 phosphorylation site [44], that has been FLAG-tagged to allow distinguishing it from the endogenous Stat3β. Stat3β was previously shown to act in a dominant negative manner to suppress the transcriptional activity of Stat3α [42]. However Stat3β can be transcriptionally active under conditions where Stat3α is not, through interaction with the N-terminal segment of c-jun, [43]. Unlike Stat3β, the S3DN protein lacks detectable DNA binding or transcriptional activity in EMSA and transient transfection reporter assays, respectively [44], and would therefore not be expected to induce Stat3β-specific effects. The S3DN protein should be able to form non-functional heterodimers with endogenous forms of Stat3α or β however, blocking their ability to enter the nucleus and/or bind DNA. Alternatively, S3DN may interfere with endogenous Stat3 activity at another level, such as the phosphorylation by JAK kinases, where S3DN may occupy the Stat3 docking sites on the cytoplasmic domains of growth factor and cytokine receptors, thereby blocking phosphorylation of endogenous Stat3α. This later possibility is less likely since we do not observe a reduced level of phospho-Stat3α in the S3DN cells compared to SRB12-p9 or Neo cells (data not shown). Also, recent evidence has emerged indicating that unphosphorylated Stat3α can drive expression of several genes, including some well known oncoproteins, through a novel mechanism that is distinct from that of phosphorylated Stat3α [47]. It therefore cannot be formally ruled out that S3DN, even though it cannot be phosphorylated, could itself have effects not involving interaction with endogenous Stats.

Although the precise mechanism of suppression of Stat3 activity by S3DN is unclear, its expression in the SRB12-p9 cells reduced binding of Stat3α to DNA (Fig. 2B) and was predicted to inhibit the constitutive Stat3 activity, thereby suppressing proliferation and possibly de-repressing apoptotic signals. To our surprise, the initial characterization of the S3DN stable transfectants indicated no obvious effects on proliferation rate or viability compared to the parental SRB12-p9 cells (Table 1). Similarly, forced overexpression of the S3WT protein did not produce an increase in proliferation rate, but rather the opposite occurred, with an approximately 2 hour increase in cell doubling time observed for 2 of the S3WT clones (Table 1). While this latter result is difficult to explain, the very short doubling time for SRB12-p9 cells (15–16 hours) suggests that further increases in proliferation rate may be limited by other intrinsic factors such as nutrient and biomolecule availability. The lack of consistent reduction in cell proliferation rate for the S3DN cells could be explained by an insufficient amount of S3DN protein expression necessary to block endogenous Stat3α activity under these culture conditions. The highest expressing S3DN clone, S3DN5, shows approximately equal signal for the Stat3 α and β bands, indicating roughly equal amounts of both proteins (Fig. 2A, middle panel, lanes labeled S3DN2 and 5). While this is a substantial increase over the wild type amount of Stat3β protein (compare levels of Stat3β between S3DN and S3WT cells in Fig. 2A, middle panel), it appears insufficient to illicit the effects on cell proliferation and/or cell viability that would be consistent with a suppression of endogenous Stat3α activity.

In an attempt to determine whether expression of S3DN could affect any Stat3α regulated cellular processes, we grew the cells in SFM, an experimental stress condition known to suppress cell growth and induce apoptosis in many cell lines. Indeed, it was only after depriving the S3DN cells of FCS that a dramatic effect was observed. Several independently selected S3DN clones underwent cell death induction when grown in SFM, which was not observed with SRB12-p9, Neo or S3WT clones. This effect was characterized by cell rounding and detachment from the plate followed by disintegration into subcellular particles, all characteristics of apoptotic cell death. This effect was quantified using the MTT cell viability assay. A reduction in cell viability for the S3DN cell lines after 2 days in SFM indicated that cell death was induced, in addition to a possible reduction in proliferation rate. In contrast, the SRB12-p9 and Neo cells remained viable and continued to proliferate, with only an approximately 2–3 hour increase in doubling time.

The induction of cell death could entirely account for the reduced viability of S3DN cells grown in SFM. However, because Stat3 is also a key regulator of cell proliferation [7-9], reduced proliferation may also contribute to this effect. Comparison of the cell cycle profiles of SRB12-p9, Neo, S3DN and S3WT cells indicated that, in FCS-containing media, the distribution of cells in the G1/0, S and G2 phases of the cell cycle was similar in all cases. One exception was the higher amount of sub-G1 DNA-containing particles for the S3DN2 cell line than for the other cell lines (12% compared to approximately 5% for the other cell lines, Fig. 4A upper row), suggesting a higher rate of apoptosis even in the presence of 10% FCS. Four days of culture in SFM resulted in an increase to 28.5% sub-G1 particles for the S3DN2 cells, but not the other cell lines. It should be noted that the data in the S3DN2 -FCS panel represents the relatively small population of S3DN2 cells remaining alive after 4 days in SFM (see corresponding panels in Fig. 3B). Surprisingly, these cells still exhibited a similar percentage of cells in the G1/0 phase to those growing in FCS-containing media, indicating that S3DN expression induces cell death, but does not cause an accumulation of cells in G1/0. However there was a reduction in the percent of cells in S phase with a proportional increase in the G2 fraction for the S3DN2 cells in SFM, indicating potential blocks at both the G1 to S phase transition and at mitosis. Thus the contribution of reduced proliferation to the overall effect of the S3DN expression on these cells is minor compared to the apoptotic effect. This conclusion is further supported by our finding that the pro-apoptotic Bcl-2 family member, Bax, is upregulated in the S3DN cells grown in SFM and this effect is accompanied by accumulation of c-PARP.

The results of the present study are consistent with an anti-apoptotic role for Stat3 in human skin SCC and are also in agreement with much of the predicted role for Stat3 derived from recent mouse skin tumorigenesis studies [21-23]. In addition, it has been demonstrated that gene therapy with Stat3β was effective in suppressing tumor growth in an *in-vivo *mouse melanoma model [40]. This effect was associated with induction of the secreted death ligand TRAIL, which could induce apoptosis and cell cycle arrest of adjacent non-transfected cells [48]. Other investigators, using a complimentary approach to assessing Stat3 function, have demonstrated that expression of the constitutively active Stat3C protein in fibroblasts can protect them from UV-induced apoptosis [41].

## Conclusion

We have demonstrated that suppression of Stat3 signaling through forced expression of the S3DN protein in human skin SCC cells blocks their growth factor- and/or other serum factor-independence. Unlike the parental SRB12-p9 cells, the S3DN cells remain viable only when cultured under optimal growth conditions, in nutrient medium supplemented with 10% FCS. The SFM culture condition is likely to provide a closer approximation to the inhospitable conditions found in tumor microenvironments *in-vivo*. This raises the exciting possibility of using S3DN as an adjunct therapy for treatment of skin SCC. We propose that delivery of the S3DN gene or protein to tumor cells could induce apoptosis directly and/or sensitize tumor cells to the apoptosis-inducing effects of cancer therapeutic agents. Future studies are planned to explore this possibility. These include assessing the effect of S3DN expression in *in-vivo *tumor models. We will begin by comparing the malignant properties of the S3DN cells with Neo control cells in mouse subcutaneous injection tumorigenicity assays. This study, together with our *in-vivo *studies in mouse skin, supports the hypothesis that Stat3 is a central regulator of apoptosis and proliferation in malignant skin cells.

## Methods

### Cell culture and generation of stable S3DN expressing SRB12-p9 cell lines

The origin and culture of the human skin SCC cell line SRB12-p9 was described previously [45]. HaCaT cells were cultured according to [49] in Dulbecco's Modified Eagles Media (DMEM)-low glucose media, supplemented with 5% fetal calf serum (FCS). Normal human epidermal keratinocytes (NHEK) cells were purchased from Clonetics (now Cambrex BioScience, Walkersville, MD) and grown in KGM-2 media (Cambrex) supplemented with hEGF (0.1 ng/ml), insulin (5.0 μg/ml), hydrocortisone (0.5 μg/ml, calcium (0.15 mM), epinephrine (5 ng/ml), transferrin (10 μg/ml), Gentamycin (50 μg/ml), Amphotericin-B (50 ng/ml) and 2 ml bovine pituitary extract. All cells were grown in a humidified atmosphere with a 5% CO2 concentration.

The pSG5-Stat3β-Y705F-FLAG expression vector was constructed by ligating the mouse Stat3β-Y705F cDNA cassette into the EcoRI site of the pSG5 expression vector [50], followed by sequential PCR-based site-specific mutagenesis using the following oligonucleotides: 5'-TCATTGATGCAGTTTGGAAACTCGAGTAACACTCTGTGAGCTGATA-3' and 5'-TATCAGCTCACAGAGTGTTACTCGAGTTTCCAAACTGCATCAATGA-3', to introduce an Xho I site immediately upstream of the stop codon at position 723. The FLAG epitope extension was created by inserting a DNA fragment, consisting of the following complimentary oligonucleotide pair: 5'-TCGAAGACTACAAAGACGATGACGATAAATAGTAGTGATGACTTGTCATCATCGTCCTTATAATCAGATCTT-3' and 5'-TCGAAAGATCTGATTATAAGGACGATGATGACAAGTCATCACTACTATTTATCGTCATCGTCTTTGTAGTCT-3', into the newly generated Xho I site. The pSG5-Stat3α-FLAG plasmid was constructed by ligating the mouse Stat3α cassette into the EcoRI site of the pSG5 expression vector, followed by sequential PCR-based site-specific mutagenesis using the following oligonucleotides: 5'-CTTCTGGTTTCAGCTCCTCGAGCATGGGGGAGGTAGCACACT-3' and 5'-AGTGTGCTACCTCCCCCATGCTCGAGGAGCTGAAACCAGAAG-3', to introduce an Xho I site immediately upstream of the stop codon. The FLAG epitope extension was created by inserting the same complimentary oligonucleotide pair used for the Stat3β construct. The Stat3β-Y705F and Stat3α cDNA cassettes were a gift from T. Schaefer (Meso Scale Discovery). The resulting plasmids were linearized with Aat II restriction enzyme and electroporated along with the bacterial neomycin phosphotransferase gene expression vector pKJ1 [51], and stably expressing cell clones were isolated essentially as previously described [45]. 5 × 10^6 ^cells suspended in 800 μl PBS were electroporated with 5 μg of linearized, purified pSG5-Stat3β-Y705F-FLAG or pSG5-Stat3α-FLAG and 0.5 μg pKJ1 with a Bio-Rad Gene Pulser set at 200 V and 960 μF. Cells were then plated at a density of approximately 1 × 10^6 ^cells/10 cm culture plate, and after 24 h subjected to neomycin selection (300 μg/ml G418 sulfate, GIBCO/BRL, Rockville, MD) for up to 14 days. Individual colonies were isolated, propagated, and divided into two aliquots, one for freezing and the other for expansion and western blotting.

### Reagents

Recombinant human interferon alpha (IFN-α) was purchased from Serotec Inc. (Raleigh, NC). Antibodies against human Stat3, Stat3 phospho-tyrosine 705, and human β-actin were supplied by Santa Cruz Biotechnology (Santa Cruz, CA). Antibodies specific for Bax, cleaved poly [ADP-ribose] polymerase (PARP) were purchased from Cell Signaling Technology (Beverly, MA). Antibody against the FLAG epitope was from Sigma-Aldrich (St. Louis, MO).

### Western blotting and electrophoretic mobility shift assay (EMSA)

Whole cell extract purification from stably transfected cells and western blotting was performed as described previously [45], with minor modifications. Total cellular protein was prepared using RIPA lysis buffer (150 mM NaCl, 10 mM Tris-HCl pH 7.5, 1 mM EDTA, 1% NP-40, 1 mM dithiothreitol) supplemented with Complete protease inhibitor cocktail (Roche, Indianapolis, IN) according to manufacturer-provided instructions. Extracted protein was quantified using the Bio-Rad Protein Assay kit (Hercules, CA). Proteins were separated by SDS acrylamide gel electrophoresis and transferred to nitrocellulose membranes (Schleicher & Schuell, Dassel, Germany). Blots were blocked with 5% milk powder for 1 h at room temperature, followed by incubation for 1 h with antibodies for Stat3, Stat3 phospho-tyrosine 705, the cleaved form of PARP, Bax and human β-actin. Blots were then washed with PBS/0.05% Tween and incubated with horseradish peroxidase-conjugated secondary antibody for 1 h at room temperature, followed by an additional 3 washes with PBS/0.05% Tween. Chemiluminescence detection was performed according to the manufacturer's instructions (Amersham Life Sciences Inc., Piscataway, NJ) followed by autoradiography.

Nuclear extracts were prepared from approximately 1 × 10^7 ^cells. Briefly, cells were trypsinized and washed with ice-cold PBS. Pelleted cells were resuspended in ice-cold buffer A containing (10 mM HEPES pH 7.9, 10 mM KCl, 1.5 mM MgCl_2_, 0.5 mM DTT, 0.2 mM PMSF, and 0.05% NP-40). After centrifugation at 1,500 g for 3 min at 4°C the pellet was resuspended in buffer C (20 mM HEPES, 420 mM NaCl, 1.5 mM MgCl2, 0.2 mM EDTA, 0.5 mM DTT, 0.2 mM PMSF, and 25% (v/v) glycerol) for 30 minutes at 4°C, followed by centrifugation at 15,000 g for 20 min at 4°C. EMSA was performed with the Gel-Shift kit (Cat No. AY1046, Panomics, Redwood, CA) according to provided protocol. Briefly, nuclear extracts (3 μg) were incubated with 10 ng biotin-labeled Stat3 response element probe (5'-GATCCTTCTGGGAATTCCTAGATC-3') derived from the c-fos gene promoter [52], in a binding buffer (both provided in the kit, final volume of 10 μl) containing 1 μg poly (dI-dC) for 40 min at 18°C. In the competitive EMSA, nuclear extracts were incubated for 5 min at room temperature with 20 ng unlabeled probe (provided by kit) prior to the addition of the biotin-labeled probe. The reactions were loaded on a 6% polyacrylamide non-denaturing PAGE gel in 0.5 × TBE buffer and electrophoresed for 1.5 h at 150 V before being transferred to BiodyneB membranes (PallGelman Lab, Ann Arbor, MI) and detected by the reagents provided with the kit.

### Cell viability and cell cycle assays

Cell viability was determined by the MTT (3- [4, 5-dimethyltiazol 2-yl]-2,5-diphenyltetraolium) bromide assay [53], essentially as previously described [54]. Briefly, cells were plated in triplicate wells (1000 cells per well) in 100 μl growth media in 96-well plates and subjected to serum starvation the following day, for the indicated durations. At the appropriate times a solution of MTT (20 μl of a 12 mM solution in PBS) was added and incubated for 1 hour at 37°C. The cells were washed gently with PBS, and 100 μl of dimethylsulfoxide was added to the wells followed by mild shaking to dissolve the MTT precipitate. Absorbance was measured for each well using a Wallac Victor3 1420 Multilabel multiwell plate reader (Perkin-Elmer) at a wavelength of 540 nm. Mean absorbance values and standard errors based on the mean (SEM) were calculated for triplicate cultures. Cell doubling time was calculated according to the following formula: doubling time (hrs) = hrs in culture ÷ (log [A540^F^/A540^I^]/log2), where A540^F ^and A540^I ^are the mean absorbance values of triplicate cultures from the MTT assay at the end and beginning of the time span measured, respectively. The cell cycle profile of control and serum starved cells was determined by cell cycle flow cytometry based on cellular DNA content, using an FACSCalibur Cell Sorter (Becton Dickenson) essentially as described previously [55]. Cells were serum-starved for the indicated durations, trypsinized, collected and pelleted together with the material floating in the medium. Cells were fixed in cold 70% ethanol, resuspended in PBS at a density of > 10^6 ^cells/ml followed by RNase A (1 mg/ml) treatment, addition of propidium iodide (20 μg/ml final concentration) and analysis by flow cytometry. The percentage of cells at different phases of the cell cycle was determined from the raw data using the ModFit LT v 3.0 software package (Verity Software House Inc.).

## Abbreviations used

NMSC, non-melanoma skin cancer; SCC, squamous cell carcinoma; Stat, signal transducer and activator of transcription; SH2, Src homology domain 2; JAKs, Janus-activated kinases; HNSCC, head and neck SCC; S3DN, Stat3β Y705F dominant negative protein; SFM, serum-free media; DMEM, Dulbecco's Modified Eagles Media; FCS, fetal calf serum; NHEK, normal human epidermal keratinocytes; hEGF, human epidermal growth factor; IFN-α, recombinant human interferon alpha; c-PARP, cleaved form of poly [ADP-ribose] polymerase; EMSA, electrophoretic mobility shift assay; TBE, Tris borate electrophoresis buffer; MTT, 3- [4, 5-dimethyltiazol 2-yl]-2,5-diphenyltetraolium; IU, international units; S3WT, Stat3α wild type protein; Neo, SRB12-p9 cells stably transfected with the empty pSG5 expression vector and the pKJ1 neomycin resistance vector

## Authors' contributions

WY carried out Western blotting, EMSA, cell viability and cell cycle analyses and contributed to the draft of the manuscript. SC and JR participated in the generation and characterization of the stably transfected cell lines. KS-C contributed to the initial study design and participated in the characterization of the stably transfected cells. JD contributed to the initial study design and to the draft of the manuscript. JC conceived of the study, coordinated the study, participated in the generation of the stably transfected cell lines and contributed to the draft of the manuscript.
